# Inferring causal networks for genomic trios with high-dimensional confounding variables

**DOI:** 10.1093/bib/bbag611

**Published:** 2026-11-05

**Authors:** Jarred M Kvamme, Audrey Qiuyan Fu

**Affiliations:** Research Computing and Data Services, University of Idaho, Moscow, ID, United States; Institute for Interdisciplinary Data Sciences, University of Idaho, Moscow, ID, United States; Department of Family Medicine and Public Health Sciences, Wayne State University School of Medicine, Detroit, MI, United States; Center for Molecular Medicine and Genetics, Wayne State University School of Medicine, Detroit, MI, United States; Institute for AI and Data Science, Wayne State University, Detroit, MI, United States

**Keywords:** principle of Mendelian randomization, causal network inference, causal structure learning, confounding, gene regulatory networks

## Abstract

Causal structure inference under the principle of Mendelian randomization (PMR) enables the study of causal relationships between two molecular phenotypes (e.g. gene expression) by leveraging genetic variants as instrumental variables to impose constraints on edge direction. In practice, inference is often affected by numerous confounding variables, such as other genes, other processes and demographic variables, making it essential to robustly account for confounders. However, existing methods have limited ability to accommodate high-dimensional confounding. Here, we introduce Mendelian randomization for genomic networks (MRGN), a regression-based method under the PMR for inferring causal models in genomic trios from individual-level data while accounting for many confounders. MRGN extends beyond the canonical mediation model to distinguish among multiple causal relationships. By prioritizing conditional independence tests given the genetic variant and confounders, MRGN can detect causal relationships between phenotypes even when their marginal association is weak. Through extensive simulations, we demonstrate improved inference accuracy, robustness, and computational efficiency over existing approaches. We further apply MRGN to data from the Genotype-Tissue Expression Consortium to investigate how genetic variants may regulate gene expression in cis and trans.

## Introduction

An important goal in biology is to understand how genes regulate one another and how genetic variation influences gene expression [1–4]. Gene regulation involves complex networks of high dimensions, which may be represented by directed networks where directed edges represent causal (or regulatory) relationships. Although the ultimate goal in biology is to reconstruct gene regulatory networks at the genome level, it is often of interest to understand the causal relationship between a molecular phenotype (e.g. the expression level) of two genes, or between two molecular phenotypes (e.g. between gene expression and DNA methylation), which may be influenced by many other variables such as other genes, other biological processes, demographic variables, etc.

To learn the (directed) causal relationship between two phenotypes, we can apply the principle of Mendelian randomization (PMR) and leverage the information in genetic variants. The PMR assumes that genotypes in a natural population are randomly assigned, thus observational omics data can be viewed as being collected from a randomized experiment [5, 6]. This implies that an edge connecting a genotype to a phenotype will always point to the phenotype, but not the other way around [7, 8]. As a result, this constraint on edge direction makes it possible to break the symmetry of association and draw inferences about the regulatory structure among phenotypes [6, 8, 9].

Methods based on the PMR framework for causal structure learning differ fundamentally from classical Mendelian randomization (MR) methods. Traditional MR focuses on estimating causal effects and depends on three key assumptions [6, 10, 11]: (i) relevance, where the genetic variant is associated with the molecular phenotype; (ii) independence, where the variant is independent of unmeasured confounders due to random allele segregation; and (iii) exclusion restriction, where the variant influences downstream phenotypes only through the intermediate phenotype, and not through other pathways. In contrast, our PMR-based framework is not designed to estimate causal effect sizes, and does not require the exclusion restriction in the same form as classical MR. Instead, it uses the genetic variant primarily to impose directional constraints on the causal graph, such that edges from molecular phenotypes to genetic variants are not allowed. For the variant to provide useful directional information, it must be associated with at least one phenotype and independent of unmeasured confounders. However, the goal is not to estimate its causal effect on the phenotype, but to leverage this association as a structural anchor for learning the relationship between the two phenotypes. Consequently, the variant does not need to be causal itself; a tag variant can serve as the anchor as long as it provides the relevant association.

Many methods have been developed under assumptions similar to the PMR [8, 12–18], although some of these methods are interested mostly in the mediation model $V \rightarrow T_{1} \rightarrow T_{2}$, where *V* is the genetic variant, $T_{1}$ and $T_{2}$ are the variables of interest. In this model, the mediator $T_{1}$ relays the regulatory information from *V* to the outcome $T_{2}$; e.g. a genetic variant regulates the expression level of a transcription factor (TF), and its reduced expression leads to its downstream target gene being under-expressed. However, other causal relationships may exist among the three variables.

We previously developed MRPC, a constraint-based causal structure learning method that uses patterns of marginal and conditional independence to distinguish five mutually exclusive basic causal models under the PMR (Fig. 1) [8, 19, 20]. They include a null model ($V \rightarrow T_{1}$ and no relationship between $T_{1}$ and $T_{2}$, denoted M0) and four non-null models: mediation (denoted M1), v-structure (or reverse causation; $V \rightarrow T_{1} \leftarrow T_{2}$, denoted M2), conditional independence (or horizontal pleiotropy; $T_{1} \leftarrow V \rightarrow T_{2}$, denoted M3), and a fully connected model ($T_{1} \leftarrow V \rightarrow T_{2}$ and $T_{1} \leftrightarrow T_{2}$, denoted M4). The true model behind M4 may be directed: the edge between $T_{1}$ and $T_{2}$ is $T_{1} \rightarrow T_{2}$ or $T_{1} \leftarrow T_{2}$, but these two directions result in the same likelihood for the trio and are therefore Markov equivalent. Without additional information, they are indistinguishable and only a bidirected/undirected edge can be inferred for M4. The primary goal of inference in this framework is the presence and direction of the $T_{1}-T_{2}$ relationship, while *V* helps distinguish among competing causal models.

**Figure 1:**
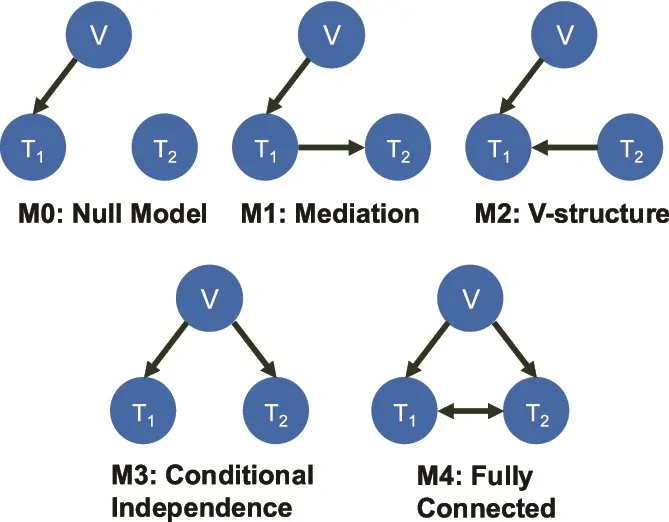
The five basic causal models for a genomic trio that may be inferred under the PMR: a genomic trio consists of a genetic variant *V* and two molecular phenotypes $T_{1}$ and $T_{2}$ (This figure is adapted from [8]).

The PMR and the five basic causal models require that no confounding variables can explain away the directed edge between $T_{1}$ and $T_{2}$, since this edge is our main interest. However, as mentioned earlier, many variables can influence the inference due to the complexity of gene regulation. A confounding variable may influence the inference in different ways. Yang *et al*. [14] proposed three types of confounding *C* for the $T_{1}-T_{2}$ edge: as a confounder ($T_{1} \leftarrow C \rightarrow T_{2}$), an intermediate variable ($T_{1} \rightarrow C \rightarrow T_{2}$), or a common child ($T_{1} \rightarrow C \leftarrow T_{2}$). Specifically, a confounder is a common parent to both $T_{1}$ and $T_{2}$ and can induce a false edge between $T_{1}$ and $T_{2}$ when unaccounted for. This is similar to $T_{1}$ and $T_{2}$ being marginally dependent in M3. An intermediate variable acts like a mediator between $T_{1}$ and $T_{2}$ and can potentially explain away a true $T_{1} \rightarrow T_{2}$ edge when this intermediate variable is accounted for in the inference, leading to a false negative conclusion. By contrast, a common child can induce a false edge between $T_{1}$ and $T_{2}$ when accounted for, leading to a false positive conclusion. This is because when the common child is accounted for in the inference, $T_{1}$ and $T_{2}$ become conditionally dependent, similar to their relationship in M2.

Accounting for confounding variables is therefore crucial in causal network inference [14, 21–24]. Two basic approaches have emerged to address latent confounding between an exposure and outcome. In the first approach, candidate confounding variables are incorporated into the graph as additional nodes in the network; MRPC and MR-GGI, for example, can infer larger networks [8, 18, 19], but the computational complexity of network inference grows super-exponentially with the number of nodes. The second approach focuses on identifying and accounting for confounders [14, 21, 22]. This approach includes all the confounders as a whole in the tests for edge presence, and does not increase the size of the network. For example, we can include the confounders when testing the $T_{1} - T_{2}$ edge in a trio. The trio remains a network of three nodes, but the tests are conditioned on these confounders. However, these methods, such as GMAC [14], focus on the mediation model.

Herein we present Mendelian Randomization for Genomic Networks (MRGN), a constraint-based method for inferring the causal structure of a genomic trio from individual-level data in the presence of potentially hundreds of confounders. Like MRPC, MRGN is also a constraint-based method such as the classic PC algorithm (named after its developers Peter Spirtes and Clark Glymour) [25], inferring graph structure from marginal and conditional independence tests. While MRGN retains from MRPC the PMR constraint and the five basic causal models, it differs from MRPC in two important aspects. First, MRGN prioritizes conditional independence tests over marginal independence tests, preserving $T_{1}-T_{2}$ relationships that may be weakened or obscured when genotypes are combined due to Simpson’s paradox. Second, rather than incorporating candidate confounders as additional network nodes, MRGN identifies confounders and adjusts for them as a whole in the independence tests. This allows large numbers of confounders to be accommodated without increasing the dimension of the inferred network and substantially reduces computational complexity. We evaluate these methodological changes through extensive simulations and apply MRGN to study cis- and trans-regulatory relationships using the whole blood data from the Genotype-Tissue Expression (GTEx) Consortium [26]. MRGN provides statistical evidence for potential causal relationships, and additional experimental validation or perturbation studies may be needed to establish causality more definitively.

## Materials and methods

### The MRGN method

The five causal models for a trio can each be described by a set of conditional and marginal dependence relations (Fig. 2a and Table 1), which may be encoded in the coefficients of two regression models that account for confounders, assuming that $T_{1}$ and $T_{2}$ are centered so that the intercept is zero:


(1) \begin{align*} T_{1} &= \beta_{11} V+\beta_{12} T_{2}+\boldsymbol{\Gamma}_{1} \boldsymbol{U}+ \epsilon_{1};\end{align*}

(2) \begin{align*}T_{2} &= \beta_{21} V+\beta_{22} T_{1}+\boldsymbol{\Gamma}_{2} \boldsymbol{U}+ \epsilon_{2},\end{align*}

**Figure 2:**
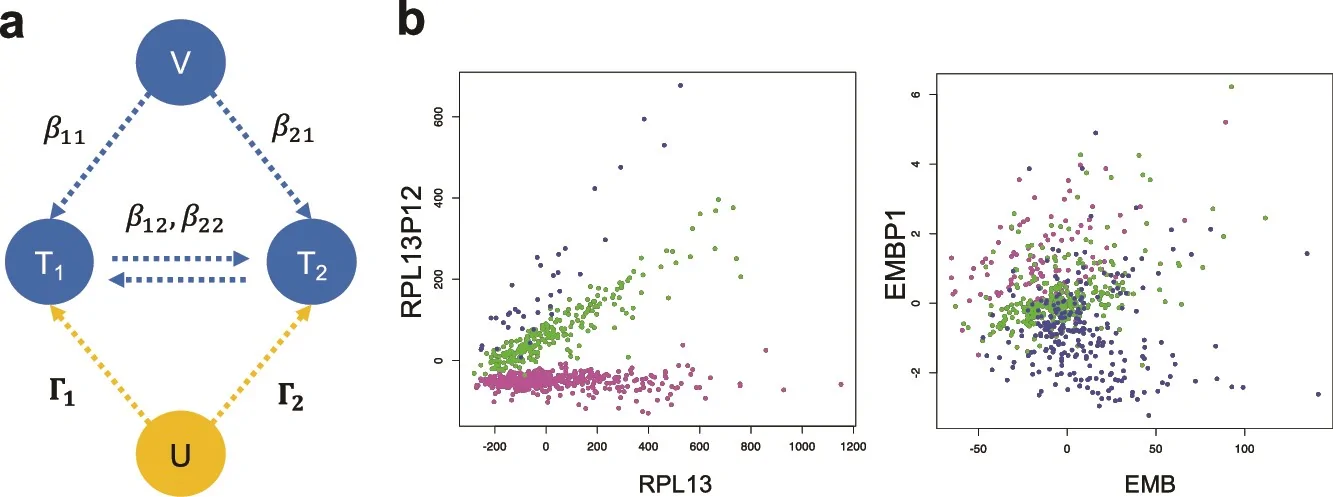
The MRGN method. (a) A genomic trio includes a genetic variant (*V*), two molecular phenotypes ($T_{1}$ and $T_{2}$), and a set of confounders ($\mathbf{U}$). (b) Two examples of Simpson’s paradox in genomic trios from the GTEx whole blood data. Each dot represents an individual, with colors indicating genotypes. Ignoring genotype, the association is weakened in the left example and disappears in the right example.

**Table 1:** Conditional and marginal independence tests in MRGN. Regression coefficients $\beta _{11}$ and $\beta _{12}$ (from Equation (1)) and $\beta _{21}$ and $\beta _{22}$ (from Equation (2)) are used to test conditional independence. The conditional test for the $T_{1}-T_{2}$ pair can be tested by $\beta _{12}$ or equivalently $\beta _{22}$, reducing the number of distinct conditional tests to three. Marginal independence is tested using Pearson correlations $r_{V,T_{1}}$ and $r_{V,T_{2}}$. The first three models each have two possible configurations

		Conditional test			Marginal test	
Null hypothesis		$\boldsymbol{T_{1}\perp} \!\!\! \perp V$	$\boldsymbol{T_{1}} \perp \!\!\! \boldsymbol{\perp T_{2}}$	$\boldsymbol{T_{2}}\perp \!\!\! \boldsymbol{\perp V}$	$\boldsymbol{V}\perp \!\!\! \perp \boldsymbol{T_{1}}$	$\boldsymbol{V} \perp \!\!\! \boldsymbol{\perp T_{2}}$
Conditioning set		$\boldsymbol{T_{2}}, \mathbf{U}$	$\boldsymbol{V}, \mathbf{U}$	$\boldsymbol{T_{1}}, \mathbf{U}$	–	–
Parameter Model	Configuration	$\boldsymbol{\beta _{11}}$	$\boldsymbol{\beta _{12}}$ (or $\boldsymbol{\beta _{22}}$)	$\boldsymbol{\beta _{21}}$	$\boldsymbol{r_{V, T_{1}}}$	$\boldsymbol{r_{V, T_{2}}}$
M0	$V \rightarrow T_{1}$ ; $T_{2}$ is singleton	*≠q 0*	*= 0*	*=0*	–	–
	$V \rightarrow T_{2}$ ; $T_{1}$ is singleton	*= 0*	*= 0*	*≠q 0*	–	–
M1	$V \rightarrow T_{1} \rightarrow T_{2}$	*≠q 0*	*≠q 0*	*= 0*	–	–
	$V \rightarrow T_{2} \rightarrow T_{1}$	* = 0*	*≠q 0*	*≠q 0*	–	–
M2	$V\rightarrow T_{1} \leftarrow T_{2}$	*≠q 0*	*≠q 0*	*≠q 0*	*≠q 0*	*=0*
	$V\rightarrow T_{2} \leftarrow T_{1}$	*≠q 0*	*≠q 0*	*≠q 0*	*=0*	*≠q 0*
M3	$T_{1} \leftarrow V \rightarrow T_{2}$	*≠q 0*	*= 0*	*≠q 0*	–	–
M4	$T_{1} \leftarrow V \rightarrow T_{2}$ ; $T_{1} \longleftrightarrow T_{2}$	*≠q 0*	*≠q 0*	*≠q 0*	*≠q 0*	*≠q 0*

where *β *s are coefficients of interest, vector $\boldsymbol{\Gamma }_{i}$ the coefficients for the confounder vector $\boldsymbol{U}_{i}$, and $\epsilon _{i}$ the random error that follows a normal distribution with mean 0 and standard deviation $\sigma _{i}$, where *i=1, 2*.

Our conditional independence tests determine whether each *β * is zero, with tests of $\beta _{12}$ and $\beta _{21}$ being equivalent because they represent the same conditional dependence. For example, in the mediation model $V \rightarrow T_{1} \rightarrow T_{2}$, *V* and $T_{2}$ are conditionally independent given $T_{1}$, corresponding to $\beta _{21}=0$, while the other coefficients are nonzero. Models M0 and M3 can similarly be distinguished using conditional independence tests alone.

In the v-structure M2, for example $V \rightarrow T_{1} \leftarrow T_{2}$, *V* and $T_{2}$ are marginally independent but conditionally dependent given $T_{1}$. Thus, all conditional associations are nonzero in both M2 and the fully connected model M4, and these models are distinguished by marginal dependence between *V* and $T_{2}$.

MRGN prioritizes conditional independence tests because the $T_{1}-T_{2}$ association within genotypes may weaken or disappear when genotypes are combined, as in Simpson’s Paradox (Fig. 2b). In contrast, MRPC tests marginal independence first and permanently removes a $T_{1}-T_{2}$ edge after a negative marginal test, potentially reducing power.

MRGN performs the standard *t* tests on the regression coefficients for conditional dependence and the standard test on Pearson correlation for marginal dependence. Since at most five tests are performed for the edges of interest, we set the significance level for each test to 0.01, providing Bonferroni control of the familywise type I error rate at 0.05 for these tests, conditional on the selected confounders. When the data are not informative enough for any of the basic models to be inferred, MRGN categorizes the trio as “other.”

### Confounder selection

We perform confounder selection before causal network inference, using a two-step procedure similar to that in the GMAC method [14]. First, we identify candidate confounders from a pool of potential confounding variables. Confounders, which are defined as parents of $T_{1}$ and $T_{2}$, should be independent of *V*. We therefore exclude variables that are marginally associated with *V*. Next, we select confounders based on their statistical association with either $T_{1}$ or $T_{2}$. Similar to Yang *et al*. [14], we test whether the regression of both $T_{1}$ and $T_{2}$ on a candidate confounder is statistically significant. Both steps allow for an additional step of the *q*-value method to control the false discovery rate (FDR) [27].

In our R implementation, we substantially improve computational efficiency in two key ways. First, we implemented parallelization in both steps, allowing for faster evaluation when the pool of confounders is large. Second, we used the R package *propagate* for correlation matrix computation by partitioning the problem into smaller, memory-efficient blocks [28], which scales more efficiently to large genomic datasets.

The overall MRGN analysis workflow, from data preprocessing through confounder identification and selection to causal model inference, is summarized in Supplementary Fig. 1.

### Simulation study

#### Simulation setup

Each simulated trio consists of a bi-allelic genetic variant *V* with three genotypes and two genes $(T_{1}, T_{2})$ connected under one of the five causal models. Nodes in a trio are generated in topological order. The genotype $V_{i}$ is simulated first under Hardy–Weinberg equilibrium with the minor allele frequency *θ *:


\begin{align*} & \Pr(V_{i} = 0) = (1-\theta)^{2},\quad \Pr(V_{i} = 1) = 2\theta(1-\theta),\quad \Pr(V_{i} = 2) = \theta^{2}. \end{align*}

Each remaining node $Y_{j}$ (a gene or a confounder) is sampled from


\begin{align*} & Y_{j} \sim N\!\left(\sum_{i\in S_{j}} \beta_{i} Y_{i},\;\sigma^{2}\right), \end{align*}

where $S_{j}$ is the parent set of $Y_{j}$, which may include *V*, the other gene, or confounders. For M4, the true edge between $T_{1}$ and $T_{2}$ can be in either direction. For simplicity, we fix all M4 trios to the configuration $T_{1} \leftarrow V \rightarrow T_{2}$ with $T_{1} \rightarrow T_{2}$.

We performed two simulations. Simulation A evaluated confounder selection and causal network inference across three effect sizes and four sample sizes. Effect sizes were defined by the effects of *V* on *T* and between the two *T*s:

- Weak: Uniform (0.1, 0.5) and Uniform (0.1, 0.3), respectively;
- Moderate: Uniform (0.5, 1) and Uniform (0.3, 0.5), respectively;
- Strong: Uniform (1, 1.5) and Uniform (0.5, 1), respectively.

The weak setting approximated the genotype–cis gene associations in the GTEx whole-blood data [26]: the median partial correlation between *V* and $T_{1}$, conditional on the true confounders, was 0.14 in the simulations, compared with a mode of *∼ *0.13 for the corresponding partial correlations conditional on selected expression-derived principal components (PCs) in GTEx. The moderate and strong settings were consistent with those used by Yang *et al*. [14].

Each trio included 1–50 confounders, one intermediate, and one common child. Confounder effects were sampled from Uniform (0, 0.3), intermediate effects from Uniform (0.05, 0.5), and common-child effects from Uniform (1, 1.5), each with a 50% probability of being negative. Confounder effects were also calibrated to expression-derived PCs in GTEx, with median absolute correlations of 0.10 in the simulated and GTEx data.

We considered sample sizes of 50, 150, 300, and 1000, fixed the residual variance at 1, and sampled the minor allele frequency from $\{0.1,0.2,0.3,0.4,0.5\}$. For each combination of effect size and sample size, we generated 20 datasets for each of the five causal models, yielding $3\times 4\times 5\times 20=1200$ trios.

Simulation B evaluated the impact of different types of confounding using four scenarios: confounders only, confounders plus one intermediate, confounders plus one common child, and all three types. We considered the same three effect sizes but fixed the sample size at 670, matching the GTEx v8 whole-blood data used in our application. For each scenario, we generated 20 datasets for each causal model and effect size, yielding $3\times 5\times 20=300$ trios.

#### Performance metrics

We evaluated three sets of recall (i.e. power) and precision to quantify the performance on the entire model of a trio, on individual edges and specifically on the $T_{1}-T_{2}$ edge.

1. On the causal model type: a trio is considered correctly inferred when its inferred model type matches its true type, thus requiring all edges to be inferred correctly.
  (3) \begin{align*} \mathrm{Recall}_{\text{model M}} &= \frac{\text{Number of correctly inferred trios}}{\text{Total number of trios of the causal model M}};\end{align*}
   
  (4) \begin{align*}&\mathrm{Precision}_{\text{model M}} \notag\\&= \frac{\text{Number of correctly inferred trios}}{\text{Total number of trios inferred to be of the causal model M}}.\end{align*}
2. On individual edges: we first calculated recall and precision based on individual edges in a single trio. Similar to our earlier work [8], we counted an edge with the wrong direction as a half correct edge.
  (5) \begin{align*} \mathrm{Recall}_{\mathrm{edges}} &= \frac{\text{Number of correct edges in inferred graph}}{\text{Total edges in true graph}};\end{align*}
   
  (6) \begin{align*}\mathrm{Precision}_{\mathrm{edges}} &= \frac{\text{Number of correct edges In inferred graph}}{\text{Total edges in inferred graph}}.\end{align*}
  We then calculated the mean metric across all trios.
3. On the $T_{1} - T_{2}$ edge (allowing for comparison with the GMAC method): the direct of this edge was ignored for this evaluation. Across all trios,
  (7) \begin{align*} &\mathrm{Recall}_{{{T_{1} - T_{2}}}} \notag\\&= \frac{\text{ Number of trios with a correctly identified } {T_{1} - T_{2}}\ \mathrm{edge}}{\text{ Total trios with a true } {T_{1} - T_{2}}\ \mathrm{edge}};\end{align*}
   
  (8) \begin{align*}&\mathrm{Precision}_{{{T_{1} - T_{2}}}} \notag\\& =\frac{\text{ Number of trios with a correctly identified } {T_{1} - T_{2}}\ \mathrm{edge}}{\text{ Total trios with an inferred } {T_{1} - T_{2}}\ \mathrm{edge}}.\end{align*}

## Results

### Simulation results

####  MRGN improves power and precision to detect edges in the presence of confounding

Before applying MRGN, we examined two approaches to confounder selection: controlling the FDR at *5%* with the *q*-value method (denote “CS-q”), and not correcting for multiple testing but instead setting the type I error rate of each individual test at *α <0.01* (denoted “CS-*α *”). We evaluated their ability to identify true confounders using precision and recall. Selection performance depended strongly on sample size but little on the simulated effect sizes (Supplementary Figs 2 and 3). CS-q was highly conservative at small sample sizes, with near-zero recall at *n=50* and *n=150*, but improved substantially with increasing sample size, reaching a precision of 0.84 and recall of 0.79 at *n=1000*. In contrast, as expected, CS-*α * selected many more covariates and consequently achieved substantially higher recall, increasing from 0.08 at *n=50* to 0.93 at *n=1000*, at the cost of low precision (0.02–0.21). Thus, CS-q and CS-*α * represent different precision-recall tradeoffs: CS-q favors a smaller, more specific set of confounders, whereas CS-*α * favors more complete confounder recovery at the expense of including many non-confounders.

Simulation A showed that MRGN reliably detected the $T_{1}-T_{2}$ edge when sufficient information was available, whereas recovery of the exact causal model was more challenging and varied across models (Tables 2 and 3). Increasing the effect size or sample size generally improved MRGN performance. With CS-q, recall for the $T_{1}-T_{2}$ edge increased from 0.257 under weak effects to 0.830 under strong effects, and from 0.194 at *n=50* to 0.844 at *n=1000*. Recall for individual causal models also generally increased with effect size and sample size. For example, as *n* increased from 50 to 1000, recall increased from 0.183 to 0.767 for M1 and from 0 to 0.633 for M4, whereas the improvement for M3 was more modest, from 0.150 to 0.333. Precision was generally high (>0.8) for M2 and M3 except under weak effects. MRGN with CS-*α * showed qualitatively similar trends, generally with somewhat lower recall and higher precision than CS-q. MRPC recovered M0 and M3 substantially better, consistent with its conservative behavior in calling edges. MR-GGI detected fewer edges overall and generally recovered M1 and M4 well, but rarely recovered the other models.

**Table 2:** Mean recall and precision (standard errors in parentheses) from Simulation A, stratified by method and effect size. NA indicates that the metric cannot be evaluated because no trios were inferred as the corresponding model. MRGN (Truth) uses the true confounders in the inference. GMAC assumes a mediation model and only infers the $T_{1}-T_{2}$ edge. Highest mean precision and recall is highlighted in each row (excluding MRGN truth)

Weak effects												
	MRGN (Truth)		MRGN (CS-q)		MRGN (CS-a)		MRPC (CS-q)		MR-GGI (CS-q)		GMAC	
	Recall	Precision	Recall	Precision	Recall	Precision	Recall	Precision	Recall	Precision	Recall	Precision
M0	0.490 (0.050)	0.605 (0.054)	0.130 (0.034)	0.265 (0.063)	0.130 (0.034)	**0.302 (0.070)**	**0.150 (0.046)**	0.300 (0.084)	0.050 (0.022)	0.263 (0.101)	–	–
M1	0.430 (0.050)	0.462 (0.052)	**0.210 (0.041)**	0.233 (0.045)	0.170 (0.038)	0.250 (0.053)	0.033 (0.023)	**0.500 (0.250)**	0.050 (0.022)	0.208 (0.083)	–	–
M2	0.010 (0.010)	0.125 (0.117)	**0.010 (0.010)**	0.167 (0.152)	**0.010 (0.010)**	**0.200 (0.179)**	0.000 (0.000)	0.000 (0.000)	0.000 (0.000)	NA	–	–
M3	0.350 (0.048)	0.814 (0.059)	0.070 (0.026)	0.538 (0.138)	**0.130 (0.034)**	**0.765 (0.103)**	0.050 (0.028)	0.750 (0.217)	0.010 (0.010)	0.500 (0.354)	–	–
M4	0.220 (0.041)	0.957 (0.043)	0.180 (0.038)	0.474 (0.081)	0.150 (0.036)	0.556 (0.096)	0.033 (0.023)	**1.000 (0.000)**	**0.300 (0.046)**	0.476 (0.063)	–	–
$T_{1}-T_{2}$ Edge	0.397 (0.028)	0.960 (0.018)	0.257 (0.025)	0.575 (0.043)	0.207 (0.023)	0.620 (0.049)	0.039 (0.014)	**1.000 (0.000)**	0.083 (0.016)	0.676 (0.077)	**0.643 (0.028)**	0.581 (0.027)
**Moderate effects**												
	**MRGN (Truth)**		**MRGN (CS-q)**		**MRGN (CS-a)**		**MRPC (CS-q)**		**MR-GGI (CS-q)**		**GMAC**	
	**Recall**	**Precision**	**Recall**	**Precision**	**Recall**	**Precision**	**Recall**	**Precision**	**Recall**	**Precision**	**Recall**	**Precision**
M0	0.740 (0.044)	0.718 (0.044)	0.370 (0.048)	0.463 (0.056)	0.360 (0.048)	**0.720 (0.063)**	**0.517 (0.065)**	0.508 (0.064)	0.030 (0.017)	0.600 (0.219)	–	–
M1	0.710 (0.045)	0.573 (0.044)	**0.550 (0.050)**	0.355 (0.038)	0.520 (0.050)	0.433 (0.045)	0.133 (0.044)	0.296 (0.088)	0.460 (0.050)	**0.605 (0.056)**	–	–
M2	0.420 (0.049)	0.977 (0.023)	**0.360 (0.048)**	0.923 (0.043)	0.250 (0.043)	**1.000 (0.000)**	0.183 (0.050)	0.524 (0.109)	0.000 (0.000)	NA	–	–
M3	0.710 (0.045)	0.947 (0.026)	0.300 (0.046)	0.857 (0.059)	0.350 (0.048)	**1.000 (0.000)**	**0.450 (0.064)**	0.771 (0.071)	0.010 (0.010)	0.500 (0.354)	–	–
M4	0.600 (0.049)	0.923 (0.033)	0.450 (0.050)	0.542 (0.055)	0.380 (0.049)	0.704 (0.062)	0.283 (0.058)	**0.850 (0.080)**	**0.600 (0.049)**	0.429 (0.042)	–	–
$T_{1}-T_{2}$ Edge	0.750 (0.025)	0.970 (0.011)	0.647 (0.028)	0.700 (0.028)	0.543 (0.029)	0.819 (0.027)	0.367 (0.036)	**0.971 (0.020)**	0.423 (0.029)	0.774 (0.033)	**0.850 (0.021)**	0.687 (0.024)
**Strong effects**												
	**MRGN (Truth)**		**MRGN (CS-q)**		**MRGN (CS-a)**		**MRPC (CS-q)**		**MR-GGI (CS-q)**		**GMAC**	
	**Recall**	**Precision**	**Recall**	**Precision**	**Recall**	**Precision**	**Recall**	**Precision**	**Recall**	**Precision**	**Recall**	**Precision**
M0	0.910 (0.029)	0.901 (0.030)	0.580 (0.049)	0.806 (0.047)	0.580 (0.049)	**0.829 (0.045)**	**0.833 (0.048)**	0.704 (0.054)	0.000 (0.000)	NA	–	–
M1	0.860 (0.035)	0.534 (0.039)	0.800 (0.040)	0.408 (0.035)	0.580 (0.049)	0.443 (0.043)	0.450 (0.064)	0.422 (0.062)	**0.810 (0.039)**	**0.551 (0.041)**	–	–
M2	0.760 (0.043)	1.000 (0.000)	**0.660 (0.047)**	0.985 (0.015)	0.500 (0.050)	**1.000 (0.000)**	0.550 (0.064)	0.635 (0.067)	0.000 (0.000)	NA	–	–
M3	0.860 (0.035)	0.977 (0.016)	0.450 (0.050)	0.918 (0.039)	0.280 (0.045)	**1.000 (0.000)**	**0.650 (0.062)**	**1.000 (0.000)**	0.010 (0.010)	**1.000 (0.000)**	–	–
M4	0.290 (0.045)	0.935 (0.044)	0.230 (0.042)	0.354 (0.059)	0.190 (0.039)	**0.373 (0.068)**	0.083 (0.036)	0.294 (0.111)	**0.340 (0.047)**	0.256 (0.038)	–	–
$T_{1}-T_{2}$ Edge	0.880 (0.019)	0.985 (0.007)	0.830 (0.022)	0.759 (0.024)	0.630 (0.028)	0.815 (0.026)	0.700 (0.034)	**0.947 (0.019)**	0.600 (0.028)	0.709 (0.029)	**0.963 (0.011)**	0.739 (0.022)

**Table 3:** Mean recall and precision (standard errors in parentheses) from Simulation A, stratified by method and sample size. NA indicates that the metric cannot be evaluated because no trios were inferred as the corresponding model. MRGN (Truth) uses the true confounders in the inference. GMAC assumes a mediation model and only infers the $T_{1}-T_{2}$ edge. Highest mean precision and recall is highlighted in each row (excluding MRGN truth)

n = 50												
	MRGN (Truth)		MRGN (CS-q)		MRGN (CS-a)		MRPC (CS-q)		MR-GGI (CS-q)		GMAC	
	Recall	Precision	Recall	Precision	Recall	Precision	Recall	Precision	Recall	Precision	Recall	Precision
M0	0.300 (0.059)	0.353 (0.067)	**0.333 (0.061)**	**0.392 (0.068)**	0.000 (0.000)	NA	0.233 (0.055)	0.359 (0.077)	0.000 (0.000)	0.000 (0.000)	–	–
M1	0.117 (0.041)	0.333 (0.103)	0.183 (0.050)	0.314 (0.078)	0.000 (0.000)	NA	**0.217 (0.053)**	**0.433 (0.090)**	0.200 (0.052)	0.324 (0.077)	–	–
M2	0.117 (0.041)	1.000 (0.000)	0.067 (0.032)	0.667 (0.192)	0.000 (0.000)	NA	**0.100 (0.039)**	**0.857 (0.132)**	0.000 (0.000)	NA	–	–
M3	0.183 (0.050)	0.846 (0.100)	0.150 (0.046)	**1.000 (0.000)**	0.000 (0.000)	NA	**0.167 (0.048)**	**1.000 (0.000)**	0.050 (0.028)	**1.000 (0.000)**	–	–
M4	0.000 (0.000)	NA	0.000 (0.000)	0.000 (0.000)	0.000 (0.000)	NA	0.000 (0.000)	NA	**0.017 (0.017)**	**0.053 (0.051)**	–	–
$T_{1}-T_{2}$ Edge	0.156 (0.027)	1.000 (0.000)	0.194 (0.029)	0.814 (0.059)	0.000 (0.000)	NA	0.206 (0.030)	**1.000 (0.000)**	0.161 (0.027)	0.690 (0.071)	**0.606 (0.036)**	0.736 (0.036)
**n = 150**												
	**MRGN (Truth)**		**MRGN (CS-q)**		**MRGN (CS-a)**		**MRPC (CS-q)**		**MR-GGI (CS-q)**		**GMAC**	
	**Recall**	**Precision**	**Recall**	**Precision**	**Recall**	**Precision**	**Recall**	**Precision**	**Recall**	**Precision**	**Recall**	**Precision**
M0	0.733 (0.057)	0.710 (0.058)	0.300 (0.059)	0.500 (0.083)	0.233 (0.055)	0.424 (0.086)	**0.600 (0.063)**	**0.679 (0.064)**	0.033 (0.023)	0.400 (0.219)	–	–
M1	0.600 (0.063)	0.400 (0.052)	0.317 (0.060)	0.211 (0.043)	0.067 (0.032)	0.200 (0.089)	0.267 (0.057)	**0.432 (0.081)**	**0.333 (0.061)**	0.408 (0.070)	–	–
M2	0.250 (0.056)	1.000 (0.000)	0.183 (0.050)	0.917 (0.080)	0.017 (0.017)	**1.000 (0.000)**	**0.300 (0.059)**	0.621 (0.090)	0.000 (0.000)	NA	–	–
M3	0.617 (0.063)	0.902 (0.046)	0.267 (0.057)	**1.000 (0.000)**	0.017 (0.017)	**1.000 (0.000)**	**0.483 (0.065)**	0.906 (0.052)	0.000 (0.000)	NA	–	–
M4	0.117 (0.041)	1.000 (0.000)	0.067 (0.032)	0.211 (0.094)	0.000 (0.000)	NA	0.133 (0.044)	**0.727 (0.134)**	**0.267 (0.057)**	0.308 (0.064)	–	–
$T_{1}-T_{2}$ Edge	0.622 (0.036)	1.000 (0.000)	0.439 (0.037)	0.653 (0.043)	0.106 (0.023)	0.905 (0.064)	0.411 (0.037)	**0.961 (0.022)**	0.306 (0.034)	0.688 (0.052)	**0.817 (0.029)**	0.681 (0.032)
**n = 300**												
	**MRGN (Truth)**		**MRGN (CS-q)**		**MRGN (CS-a)**		**MRPC (CS-q)**		**MR-GGI (CS-q)**		**GMAC**	
	**Recall**	**Precision**	**Recall**	**Precision**	**Recall**	**Precision**	**Recall**	**Precision**	**Recall**	**Precision**	**Recall**	**Precision**
M0	0.833 (0.048)	0.847 (0.047)	0.333 (0.061)	0.541 (0.082)	0.550 (0.064)	**0.733 (0.066)**	**0.667 (0.061)**	0.571 (0.059)	0.017 (0.017)	0.200 (0.179)	–	–
M1	0.817 (0.050)	0.516 (0.051)	**0.617 (0.063)**	0.333 (0.045)	0.567 (0.064)	0.374 (0.051)	0.133 (0.044)	0.286 (0.085)	0.517 (0.065)	**0.477 (0.062)**	–	–
M2	0.417 (0.064)	0.926 (0.050)	0.283 (0.058)	0.850 (0.080)	0.200 (0.052)	**1.000 (0.000)**	**0.333 (0.061)**	0.526 (0.081)	0.000 (0.000)	NA	–	–
M3	0.733 (0.057)	0.898 (0.043)	0.317 (0.060)	0.679 (0.088)	0.383 (0.063)	**1.000 (0.000)**	**0.500 (0.065)**	0.833 (0.062)	0.000 (0.000)	0.000 (0.000)	–	–
M4	0.367 (0.062)	0.846 (0.071)	0.167 (0.048)	0.345 (0.088)	0.117 (0.041)	0.389 (0.115)	0.267 (0.057)	**0.571 (0.094)**	**0.350 (0.062)**	0.304 (0.055)	–	–
$T_{1}-T_{2}$ Edge	0.806 (0.029)	0.980 (0.012)	0.622 (0.036)	0.700 (0.036)	0.578 (0.037)	0.860 (0.032)	0.489 (0.037)	**0.936 (0.025)**	0.439 (0.037)	0.775 (0.041)	**0.828 (0.028)**	0.645 (0.031)
**n = 1000**												
	**MRGN (Truth)**		**MRGN (CS-q)**		**MRGN (CS-a)**		**MRPC (CS-q)**		**MR-GGI (CS-q)**		**GMAC**	
	**Recall**	**Precision**	**Recall**	**Precision**	**Recall**	**Precision**	**Recall**	**Precision**	**Recall**	**Precision**	**Recall**	**Precision**
M0	0.850 (0.046)	0.911 (0.038)	0.450 (0.064)	0.711 (0.074)	**0.483 (0.065)**	**0.725 (0.071)**	NA	NA	0.017 (0.017)	0.200 (0.179)	–	–
M1	0.917 (0.036)	0.632 (0.052)	**0.767 (0.055)**	0.422 (0.047)	0.717 (0.058)	0.417 (0.049)	NA	NA	0.667 (0.061)	**0.800 (0.057)**	–	–
M2	0.583 (0.064)	0.946 (0.037)	**0.583 (0.064)**	**0.921 (0.044)**	0.500 (0.065)	0.882 (0.055)	NA	NA	0.000 (0.000)	NA	–	–
M3	0.850 (0.046)	0.981 (0.019)	0.333 (0.061)	0.870 (0.070)	**0.433 (0.064)**	**0.963 (0.036)**	NA	NA	0.000 (0.000)	NA	–	–
M4	0.767 (0.055)	0.958 (0.029)	0.633 (0.062)	0.559 (0.060)	0.650 (0.062)	**0.619 (0.061)**	NA	NA	**0.750 (0.056)**	0.455 (0.050)	–	–
$T_{1}-T_{2}$ Edge	0.917 (0.021)	0.959 (0.015)	0.844 (0.027)	0.707 (0.031)	0.817 (0.029)	0.735 (0.031)	NA	NA	0.483 (0.037)	**0.737 (0.041)**	**0.933 (0.019)**	0.672 (0.030)

At the edge level, MRGN generally detected the $T_{1}-T_{2}$ edge with higher recall than MRPC, while its performance relative to GMAC depended on the amount of information available (Tables 2 and 3). GMAC had substantially higher recall under weak effects and at small sample sizes, but the difference narrowed as effect size and sample size increased, with MRGN maintaining comparable or higher precision. Because the GMAC mediation test is a special case of MRGN, these results suggest that GMAC is more sensitive for detecting a $T_{1}-T_{2}$ relationship under limited information, whereas MRGN provides greater resolution of the underlying causal structure.

To better illustrate the behavior of MRGN in causal-model inference, we aggregated the confusion matrices across effect sizes and sample sizes (Supplementary Table 1). For MRGN with CS-q, inferred M1 trios were a mixture of all five true models, whereas inferred M4 trios consisted mainly of true M3 and M4. These difficulties were not unique to MRGN. Although MRPC was more conservative in assigning causal models, it also had difficulty distinguishing M1 from M4: 51 (54%) of the 95 inferred M1 trios were true M4.

The distinction between M1 and M4 and that between M3 and M4 depend on the outcome of a single conditional independence test. Thus, these two patterns illustrate the limitation of hypothesis testing in constraint-based inference. A true M4 may be inferred as M1 when the null hypothesis is not rejected because of insufficient statistical power. Conversely, a true M3 may be inferred as M4 when the null hypothesis of no $T_{1}-T_{2}$ edge is incorrectly rejected. Thus, both insufficient evidence for a true edge and false-positive evidence for an absent edge can affect discrete causal-model assignment.

We further evaluated the runtime for all methods on CPUs of a shared computing cluster. MRGN was computationally efficient: confounder selection required 16 min in the largest setting examined (1000 covariates and 1000 trios), and causal network inference required a median of 0.0056 s per trio (Supplementary Fig. 4). For causal network inference, MRGN was approximately twice as fast as MR-GGI, six times faster than MRPC, and 100 times faster than GMAC (Supplementary Fig. 5).

#### The impact of confounding variables on causal network inference

We examined how errors in confounder selection affected MRGN inference. False positive confounders had relatively little effect on edge precision, although recall varied with the selection method and simulation setting (Supplementary Fig. 6). In contrast, increasing numbers of false negative confounders generally reduced edge recall, with the effect being most pronounced at smaller sample sizes (Supplementary Fig. 7).

The importance of confounding was also evident in comparisons with GMAC. In our simulations, GMAC had substantially higher type I error rates for the $T_{1}-T_{2}$ edge than previously reported by Yang *et al*. [14]. We attributed this difference to our more challenging simulation design, which included substantially more confounders and a much larger candidate pool. Repeating the GMAC simulations with increasing numbers of confounders reproduced the published type I error rate when only two confounders were present, but showed progressively inflated type I error as the number of confounders and candidate pool size increased (Supplementary Fig. 8).

We further examined the impact of other types of confounding on MRGN inference in Simulation B. Including an intermediate among the selected confounders had relatively little effect on either causal-model recovery or $T_{1}-T_{2}$ edge detection (Fig. 3; Supplementary Table 2). In particular, although adjustment for an intermediate could potentially attenuate the association between $T_{1}$ and $T_{2}$, we did not observe a substantial reduction in $T_{1}-T_{2}$ edge recall. In contrast, the common child was incorrectly selected as a confounder in 66% of the trios, and including this variable substantially reduced model recall for M0 and M3. This is expected because of the association between the two parent nodes induced by the common child, leading to a false positive $T_{1}-T_{2}$ edge. Nevertheless, MRGN maintained reasonable recall for the $T_{1}-T_{2}$ edge even when both an intermediate and a common child were present.

**Figure 3:**
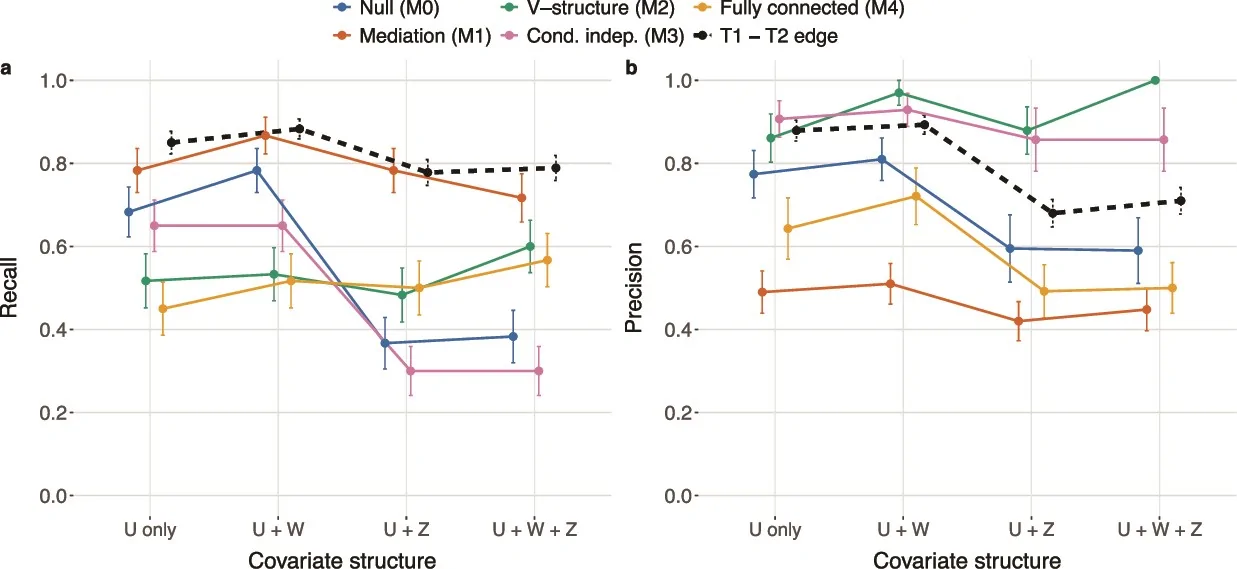
Recall and precision of MRGN with CS-q in Simulation B under different covariate structures. (a) Recall and (b) precision for the five causal models and the $T_{1}-T_{2}$ edge, aggregated across effect sizes. *U*, *W*, and *Z* denote multiple confounders, an intermediate variable, and a common child, respectively. Dots show mean recall or precision, and error bars show one standard error.

### Application to GTEx data

The GTEx consortium has identified numerous expression quantitative trait loci (eQTLs) associated with gene expression across human tissues [26]. Many eQTLs are associated with both cis- and trans-genes, motivating studies of whether cis-genes mediate their effects on trans-genes [14]. We previously applied MRPC across GTEx tissues to identify diverse causal models among eQTLs and their associated cis- and trans-genes, including protein-coding genes and long non-coding RNAs (lncRNAs) [20]. Here, we use MRGN to further explore these regulatory relationships in GTEx whole blood. Because the trios are defined by prior eQTL-gene associations, the inferred structures are interpreted as exploratory causal models conditional on this selection.

We applied the preprocessing and normalization pipeline in our previous study to whole blood data from 670 GTEx v8 samples [20]. Gene expression values were residuals from PEER normalization [29], with all GTEx-reported covariates included as known covariates: the top five genotype-derived PCs to account for population structure, sex, age, PCR batch, and platform. Each trio consisted of an eQTL and its cis- and trans-target genes of highest association, where cis-genes were located within 1 Mb of the eQTL and trans-genes were located more than 1 Mb away or on a different chromosome. After preprocessing, 8823 trios were identified. Restricting the analysis to protein-coding genes and long noncoding RNAs (lncRNAs) to reduce transcript mismapping yielded a final set of 3248 trios (Supplementary Table 3). When comparing our method to GMAC, we imputed missing genotypes using multiple correspondence analysis implemented in the *missMDA* R package [30], since GMAC cannot accommodate missing genotype values.

Since the sample size 670 is much smaller than the number of genes (*∼ *56 000) in the genomewide expression data, principal component analysis (PCA) of the PEER-normalized expression data yielded 670 PCs (with the last PC being essentially zero) as candidate confounders. Using CS-q with *q<0.05*, MRGN selected on average 30 confounders per trio (SD = 6.79) (Fig. 4a). Using CS-*α * with *α <0.01*, MRGN selected on average 51 confounders per trio (SD = 6.38) (Supplementary Fig. 9). These PCs represent potential effects of genes outside the gene pair in each trio. Although the PCs were derived from all genes, excluding the two genes in a given trio would have negligible impact on the resulting PCs.

**Figure 4:**
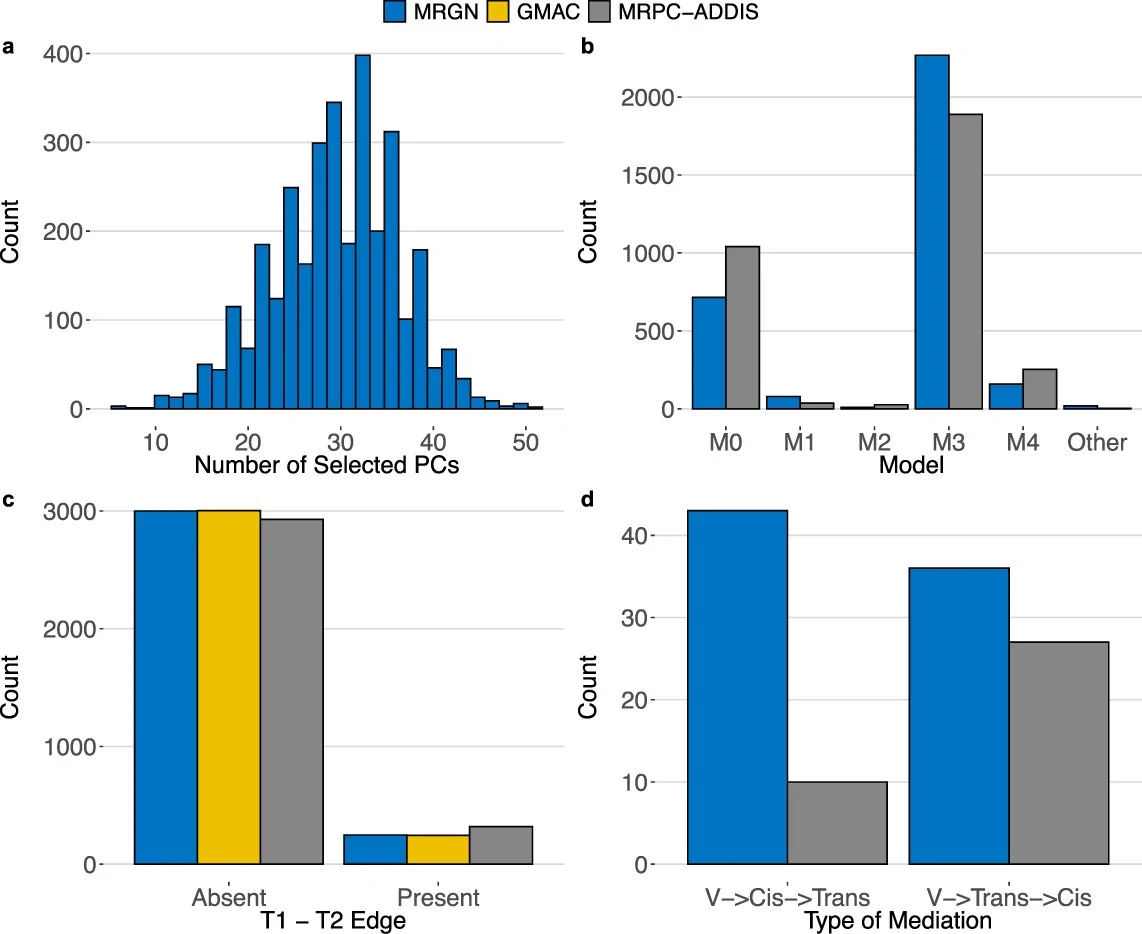
Comparative analysis of MRGN with CS-q, MRPC [8] and GMAC [14] on GTEx human whole blood trios. We applied MRGN with CS-q, GMAC, and MRPC (with ADDIS for FDR control) to genomic trios. (a) Histogram of the number of PCs selected as confounders per trio. (b) Count of inferred causal models (M1–M4 and Other) comparing MRGN and MRPC. (c) Counts of trios with the $T_{1} - T_{2}$ edge inferred as absent or present by the three methods. (d) Counts of two types of mediation models (via cis-gene versus trans-gene) inferred by MRGN and GMAC.

####  MRGN identified more mediation and conditional independence trios

Following confounder selection with CS-q, MRGN identified 715 trios as the null model, 79 as mediation, 9 as v-structure, *2268* as conditionally independent, 158 as fully connected, and 19 as “other” (Fig. 4b). With CS-*α *, these numbers are *911* null, *96* mediation, *9* v-structure, *2060* conditionally independent, *119* fully connected, and *53* “other” (Supplementary Fig. 9). Focusing on the results from CS-q, MRGN identified more than twice mediation trios and 379 additional conditional independence trios than MRPC [20], although the total number of inferred $T_{1}-T_{2}$ edges was similar across methods (Fig. 4c). As in MRPC, MRGN distinguished two mediation models (Fig. 4d): cis-gene mediation ($V \rightarrow T_{1} \rightarrow T_{2}$) and trans-gene mediation ($V \rightarrow T_{2} \rightarrow T_{1}$). Among the 79 mediation trios, 43 (54%) involved cis-gene mediation and 36 (46%) involved trans-gene mediation (Fig. 4d). In comparison, MRPC identified 10 cis-gene mediation trios and 27 trans-gene mediation trios.

Nine cis-gene mediation trios and 12 trans-gene mediation trios were identified by both methods. Thus, MRGN recovered nearly all cis-gene mediation trios identified by MRPC, whereas the agreement was lower for trans-gene mediation trios. Examination of trios classified as mediation by MRGN but not MRPC indicated that these discrepancies were primarily driven by differences in confounder selection. For example, for the trio consisting of rs117667029, *METTL26*, and *PTMS*, MRGN identified 37 confounders and inferred mediation (rs117667029 *→ *  *METTL26*  *→ *  *PTMS*), whereas MRPC identified only three confounders and inferred conditional independence (*METTL26*  *← * rs117667029 *→ *  *PTMS*). Repeating the MRGN analysis with the same three confounders also yielded conditional independence, confirming that accurate confounder selection is critical for consistent causal network inference.

To assess the robustness and uncertainty of the inferred mediation trios, we applied baycn [31], a Bayesian directed acyclic graph method that estimates posterior probabilities for edge presence and direction under the PMR constraint, to each trio together with its selected confounders (Supplementary Table 4). Unlike MRGN, baycn treats the selected confounders as network nodes and infers their edge directions rather than assuming that they point to the genes. We ran baycn for 4 million iterations with 20% burn-in and thinning every 8000 iterations. An edge is considered presence if the edge absence probability is *<0.5*, and an edge is considered directed if the absolute difference between the probabilities for the two directions is *>0.2*. Thus, an M4 trio classified from the baycn results may contain a directed $T_{1}-T_{2}$ edge. Among the 43 cis-gene mediation trios, baycn inferred 15 (35%) as the same mediation model, 28 (65%) as M4, and one as M3. Among the 36 trans-gene mediation trios, 9 (25%) were inferred as the same mediation model and 23 (64%) as M4, with the remaining six assigned to other models. Considering only the directed $T_{1}-T_{2}$ edge, MRGN and baycn agreed for 30 (70%) cis-gene and 24 (67%) trans-gene mediation trios. Thus, the predominant discrepancy was between M1 and M4, consistent with the M1–M4 confusion observed in the simulations. On the other hand, among the 17 mediation trios identified only by MRPC, baycn inferred a $T_{1}-T_{2}$ edge for only 7 (41%), consistent with the higher recall of MRGN observed in the simulations.

####  MRGN is robust to model misspecification in inference of mediation

We further investigated the effect of inference model misspecification on mediation inference using 192 mediation trios identified by GMAC in whole blood. Four scenarios were considered, ranging from models using the true confounders to models omitting many moderate-to-strong confounders (see Supplementary Text 1).

Recall remained *∼ *93% when the inference model included most influential confounders, even if a few weak confounders were incorrectly included or omitted. In contrast, omitting many moderate-to-strong confounders reduced recall to *∼ *70%, demonstrating that retaining influential confounders is critical for accurate causal network inference.

#### Biological interpretation

We formulated genomic trios to discover possible causal relationships among genes selected for their strong associations with the eQTL, rather than known regulatory relationships. Most selected genes are not TFs, and many are relatively understudied lncRNAs. Accordingly, only four out of 74 unique mediator genes are TFs represented in CollecTRI [32], a curated database of high-confidence TF-target interactions, and none of the gene pairs in the inferred mediation trios are reported TF-target pairs. Nevertheless, literature evidence supports or suggests biological mechanisms for several inferred relationships:

- chr1:25253604_G_A *→ *  *TMEM14C* (trans-gene on chr6) *→ *  *RHCE* (cis-gene), with Pearson correlations $r_{V,\mathrm{cis}}=-0.05$, $r_{V,\mathrm{trans}}=-0.22$, and $r_{\mathrm{cis},\mathrm{trans}}=0.16$ (classified as M4 by baycn, but with $T_{\mathrm{trans}}\rightarrow T_{\mathrm{cis}}$). Both genes are involved in erythroid maturation. TMEM14C, a mitochondrial inner-membrane transporter, controls intracellular heme levels [33]; heme regulates GATA1 activity and stability [34], and GATA1 in turn transactivates *RHCE* [35, 36]. The heme-GATA1 feedback loop is well established in erythroid biology [37], providing a plausible mechanism for the inferred *TMEM14C**→ **RHCE* relationship.
- chr5:122044080_C_T *→ *  *EFEMP1* (trans-gene on chr2) *→ *  *LOX* (cis-gene), with $r_{V,\mathrm{cis}}=0.03$, $r_{V,\mathrm{trans}}=0.19$, and $r_{\mathrm{cis},\mathrm{trans}}=0.39$ (baycn inferred the same model). EFEMP1 paralogs EFEMP2 (a.k.a. fibulin-4 or FBLN4) and FBLN5 (a.k.a. fibulin-5) have direct mechanistic relationships with the LOX family: EFEMP2 binds LOX and recruits it for elastin crosslinking, while FBLN5 interacts with several LOX-family proteins [38, 39]. As EFEMP1 functions in the same extracellular-matrix assembly module, the inferred edge suggests a potential role for *EFEMP1* in *LOX* regulation.
- chr17:19867907_C_T *→ *  *ULK2* (cis-gene) *→ *  *GSDMC* (trans-gene on chr8), with $r_{V,\mathrm{cis}}=-0.11$, $r_{V,\mathrm{trans}}=0.18$, and $r_{\mathrm{cis},\mathrm{trans}}=-0.12$ (baycn inferred the same model). ULK1, a close paralog of ULK2, directly phosphorylates and activates GSDMA, a paralog of GSDMC, during starvation-induced pyroptosis [40]. Gasdermins including GSDMA and GSDMC are also linked to autophagy [41], while ULK2 is a core autophagy-initiating kinase. The inferred *ULK2**→ **GSDMC* edge therefore suggests a potential interaction between these two gene families.

## Discussion

In summary, we have developed MRGN to improve the power and accuracy of detecting key causal relationships in diverse genomic trios, particularly in the presence of multiple confounding variables. Our method adopts an extended interpretation of the PMR [8] and prioritizes conditional independence tests to identify five basic causal models. A major advantage of our approach is the reduction of the multiple testing burden; by adjusting for selected confounders within a regression framework, MRGN can incorporate a large number of confounders without the need to infer a graph larger than three nodes.

A general challenge in causal structure learning is that statistical support for individual edges is continuous, whereas interpreting the inferred structure often requires deciding whether an edge is present and, if so, its direction. Constraint-based methods make these discrete decisions through thresholds in hypothesis testing, while score-based and Bayesian methods may similarly require thresholds on edge weights or edge inclusion probabilities. Thus, converting continuous statistical evidence into discrete edge decisions generally requires a prespecified criterion. In constraint-based methods, failure to reject the null hypothesis may indicate either genuine edge absence or insufficient statistical support for edge presence. This leads to difficulty distinguishing models, particularly M1 and M4, which differ by a single conditional independence test, as we observe in the simulations and GTEx analysis. As an alternative, equivalence testing may provide stronger evidence for edge absence, but requires specifying the equivalence bounds, which may depend on the phenotype, application, and scale of the data. Thus, equivalence testing does not eliminate the need for a threshold, but instead requires a threshold to be specified for a different quantity.

While our simulations and real data analysis demonstrate the flexibility of MRGN in estimating causal relationships, the method is currently limited to trios containing a genetic variant that acts as an instrumental variable. In larger networks, candidate gene pairs may first be identified based on biological interest, observed associations, or other prior information. Genetic variants associated with one or both genes can then be identified and included as anchors for causal inference, as in our GTEx analysis. Overlapping trios may produce relationships that are not globally consistent, and assembling them into a coherent network requires additional methodology. We are currently extending MRGN to a general method that jointly infers larger networks and incorporates potential intermediate variables and common children as additional network nodes, while confounders are accounted for in the inference as in MRGN.

**Key Points**

- Mendelian Randomization for genomic networks (MRGN) is a regression-based method for inferring causal structures in genomic trios from individual-level data while accounting for many confounders. A genomic trio consists of two molecular phenotypes and a genetic variant.
- MRGN leverages the Principle of Mendelian randomization, which uses genetic variants as instrumental variables to impose constraints on edge directions in the inference of causal network structure.
- By prioritizing conditional independence tests given the genetic variant and confounders, MRGN can detect causal relationships between phenotypes even when their marginal association is weak.

## Supplementary Material

Supplementary_materials_bbag611

## Data Availability

Data from The GTEx Project version 8 were obtained from dbGaP (http://www.ncbi.nlm.nih.gov/gap) through the following accession number: phs000424.v8.pht002741.v8.p2. Open-source R packages used in this manuscript include MRGN; https://github.com/audreyfulab/MRGN, MRPC (version 3.1.0); https://cran.r-project.org/web/packages/MRPC/index.html, and GMAC (version 3.1; https://cran.r-project.org/web/packages/GMAC/index.html. R code for simulations and applications with results is available at https://github.com/audreyfulab/MRGN_Manuscript_Repo. Additional results are also included in the supplementary material.
