# Supplementary material for: Inferring causal networks for genomic trios with high-dimensional confounding variables
Source: Brief Bioinform. 2026 Nov 5;27(6):bbag611. doi: 10.1093/bib/bbag611 (PMC13738103; doi:10.1093/bib/bbag611)
Supplement: Supplementary file 1 [file supplementary_materials_bbag611.zip › Supplementary_Figures_bbag611.pdf]

Inferring causal networks for genomic trios with high-dimensional confounding variables

Supplementary figures

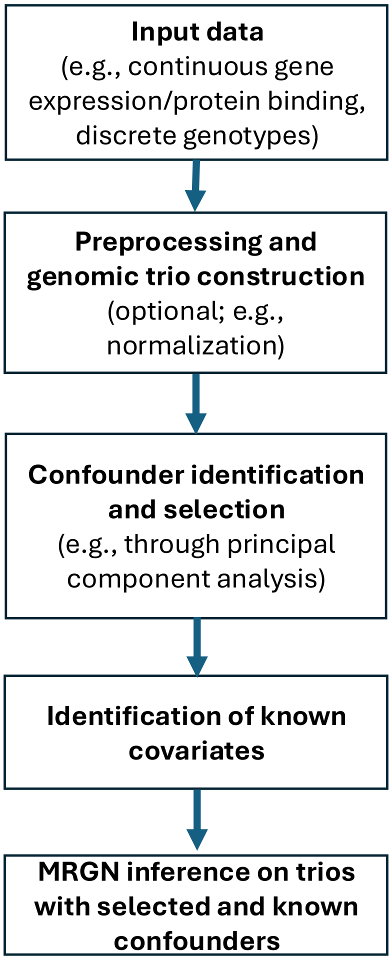

Supplementary Figure 1: The flowchart of using MRGN for data analysis.

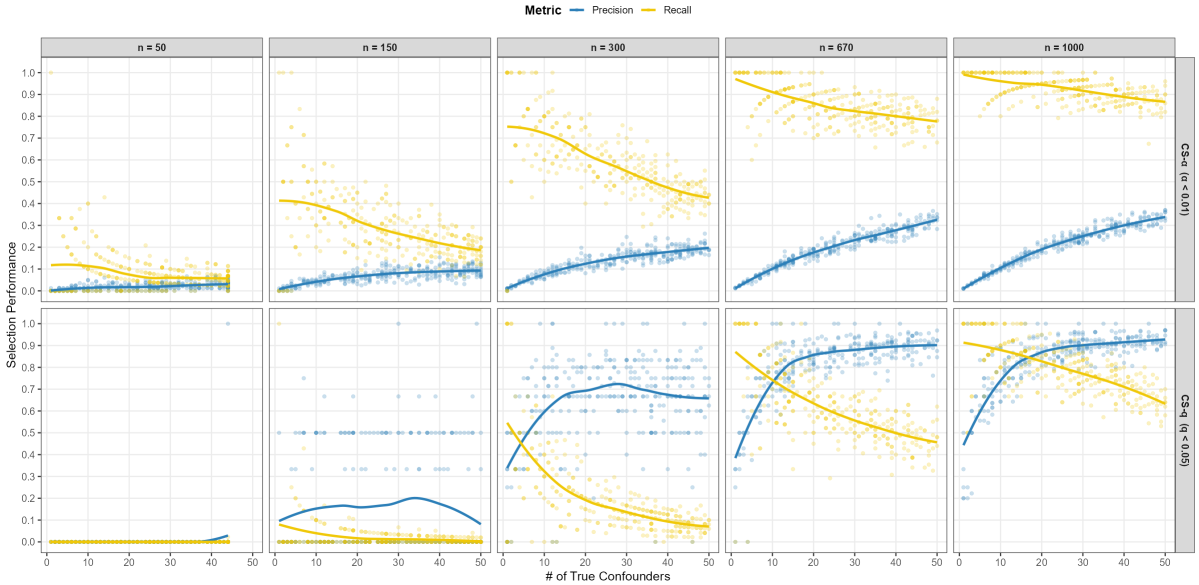

Supplementary Figure 2: Precision and recall of confounder selection on simulated data by two multiple-testing correction methods: CS-q and CS-α, stratified by sample size. Each dot is a simulated data set.

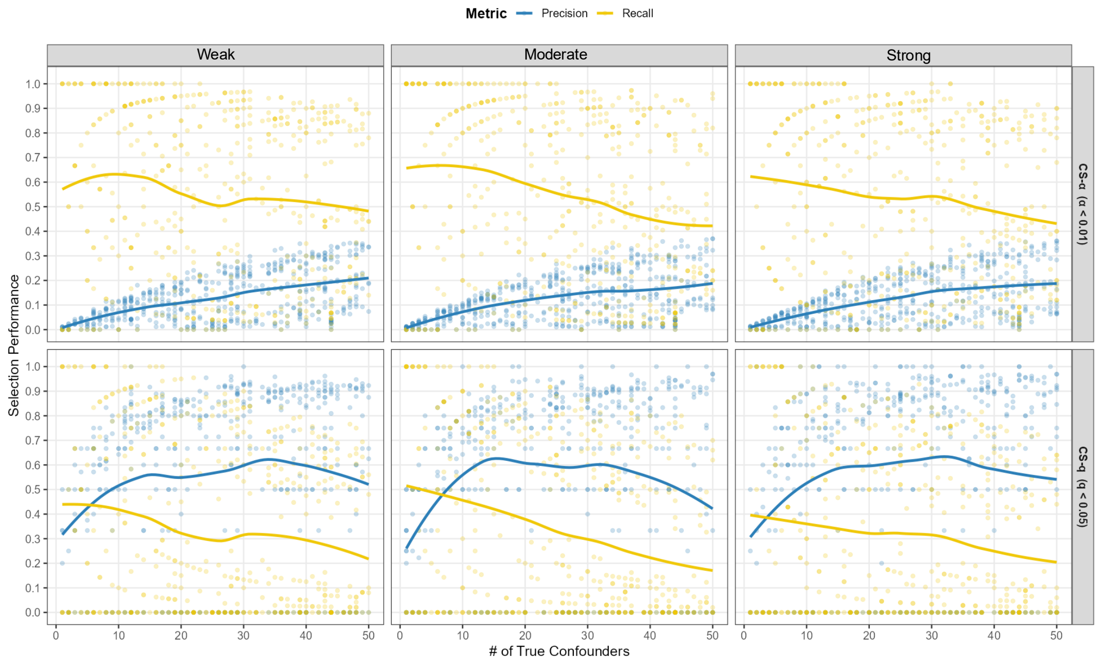

Supplementary Figure 3: Precision and recall of confounder selection on simulated data by two multiple-testing correction methods: CS-q and CS-α, stratified by effect size. Each dot is a simulated data set.

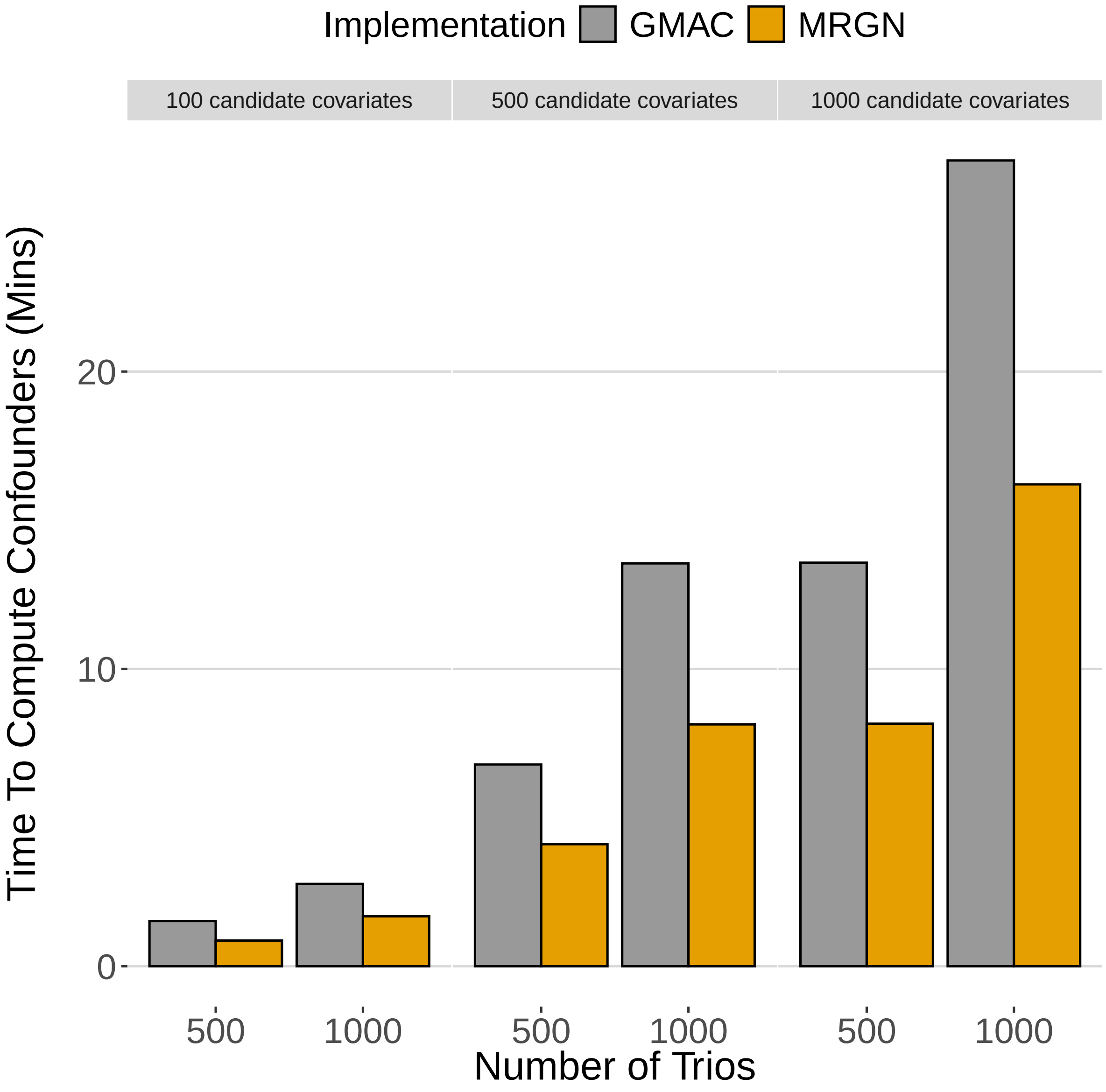

Supplementary Figure 4: Comparison of computation times between our implementation of the CS-q method for confounder selection and that of the GMAC method. Computation times are shown across different numbers of input trios and sizes of the candidate confounder pool.

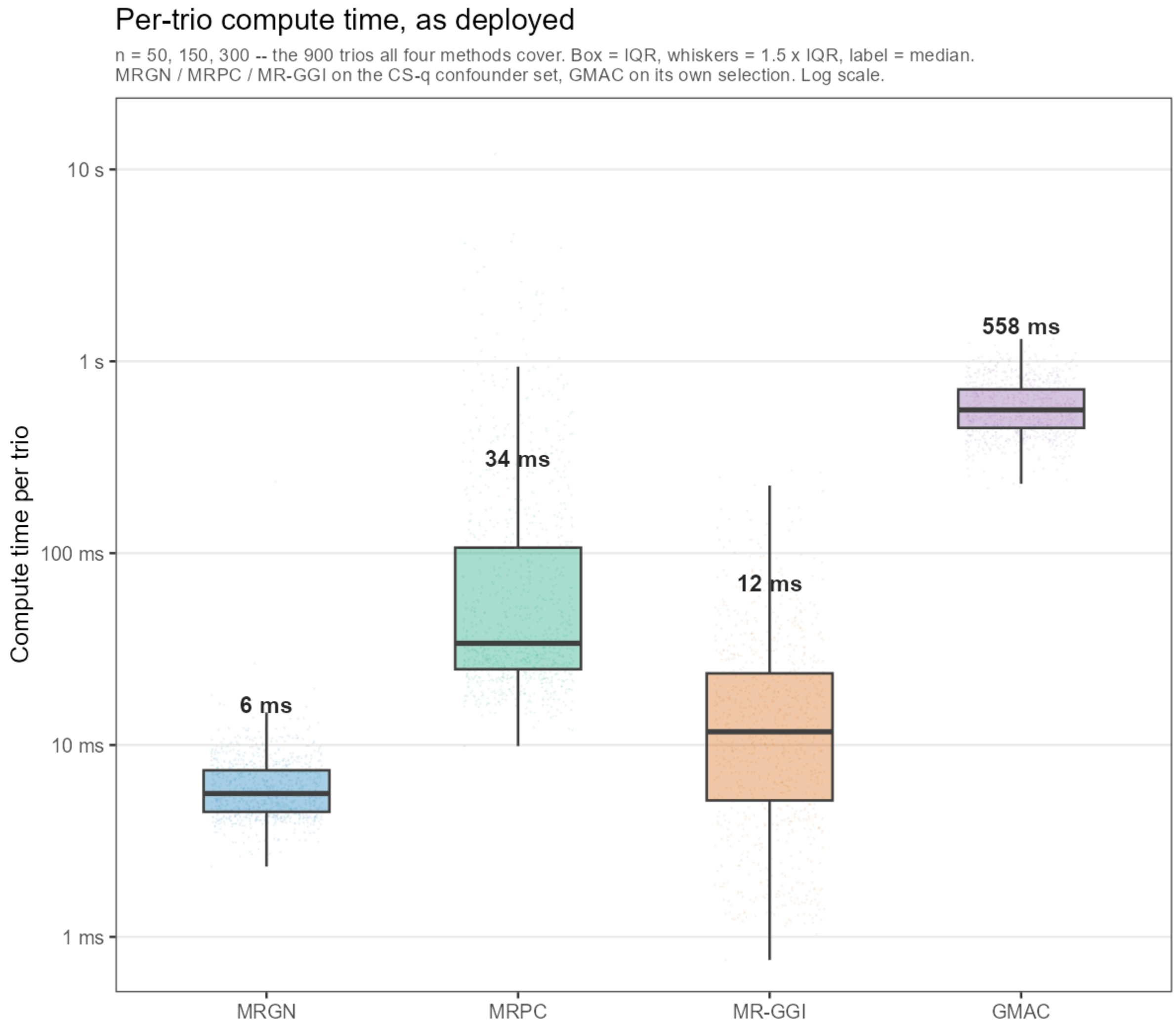

Supplementary Figure 5: Boxplots of runtime for causal network inference after confounder selection from different methods on simulated trios.

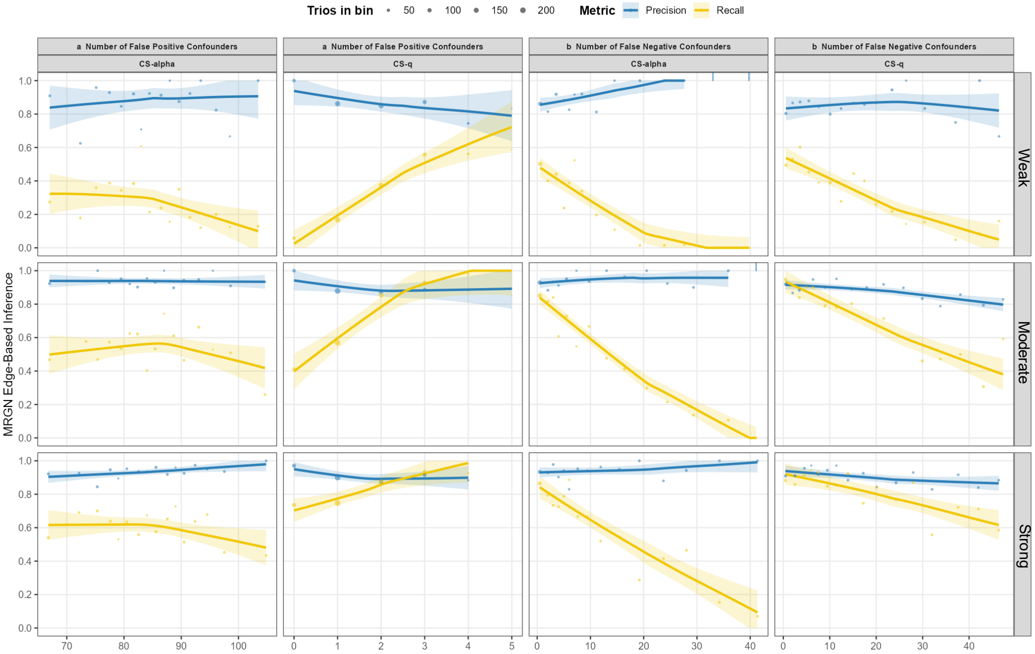

Supplementary Figure 6: Relationship between confounder selection and $T_1 - T_2$ edge inference, stratified by false positive/negative confounders and effect size.

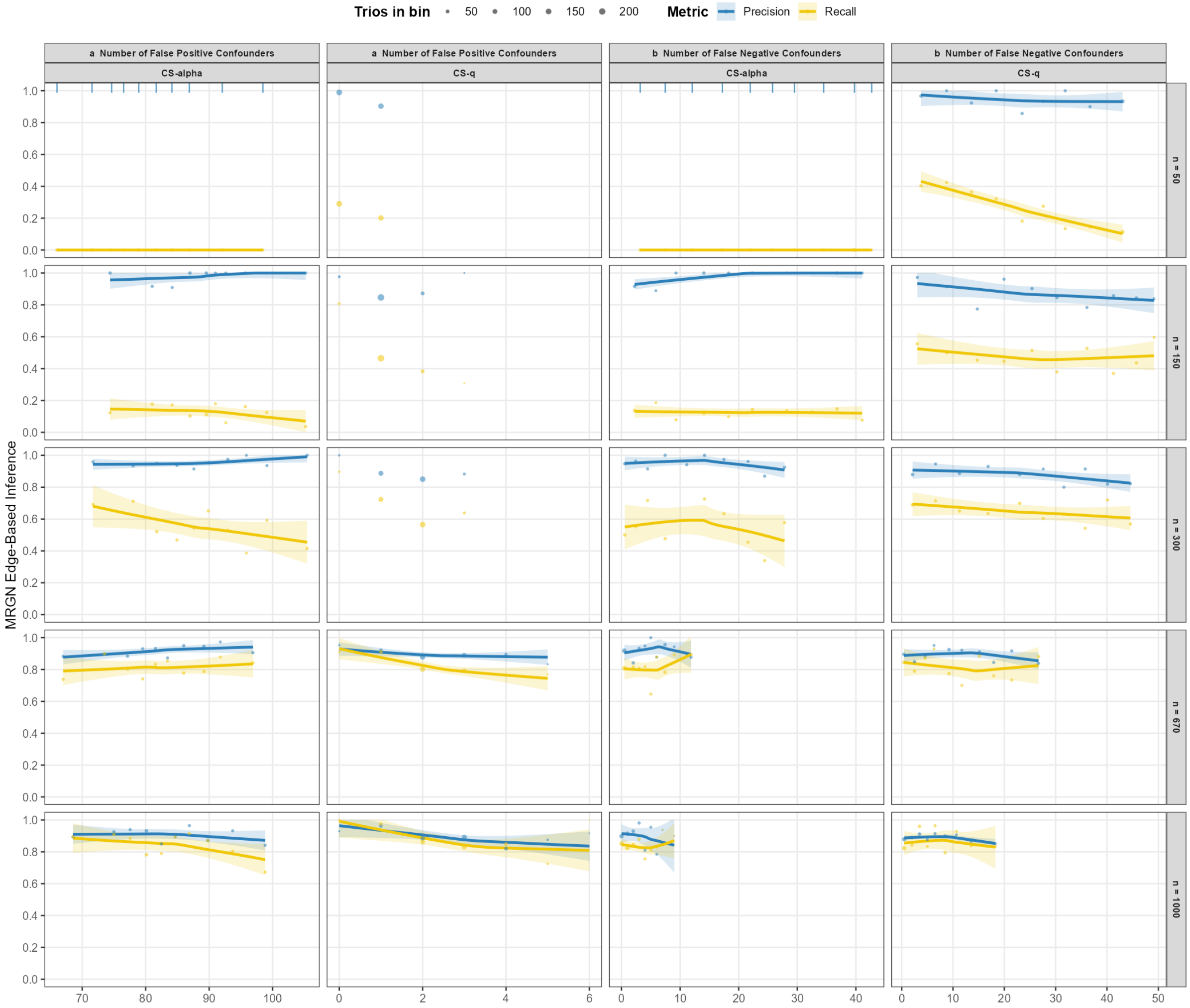

Supplementary Figure 7: Relationship between confounder selection and $T_1 - T_2$ edge inference, stratified by false positive/negative confounders and sample size.

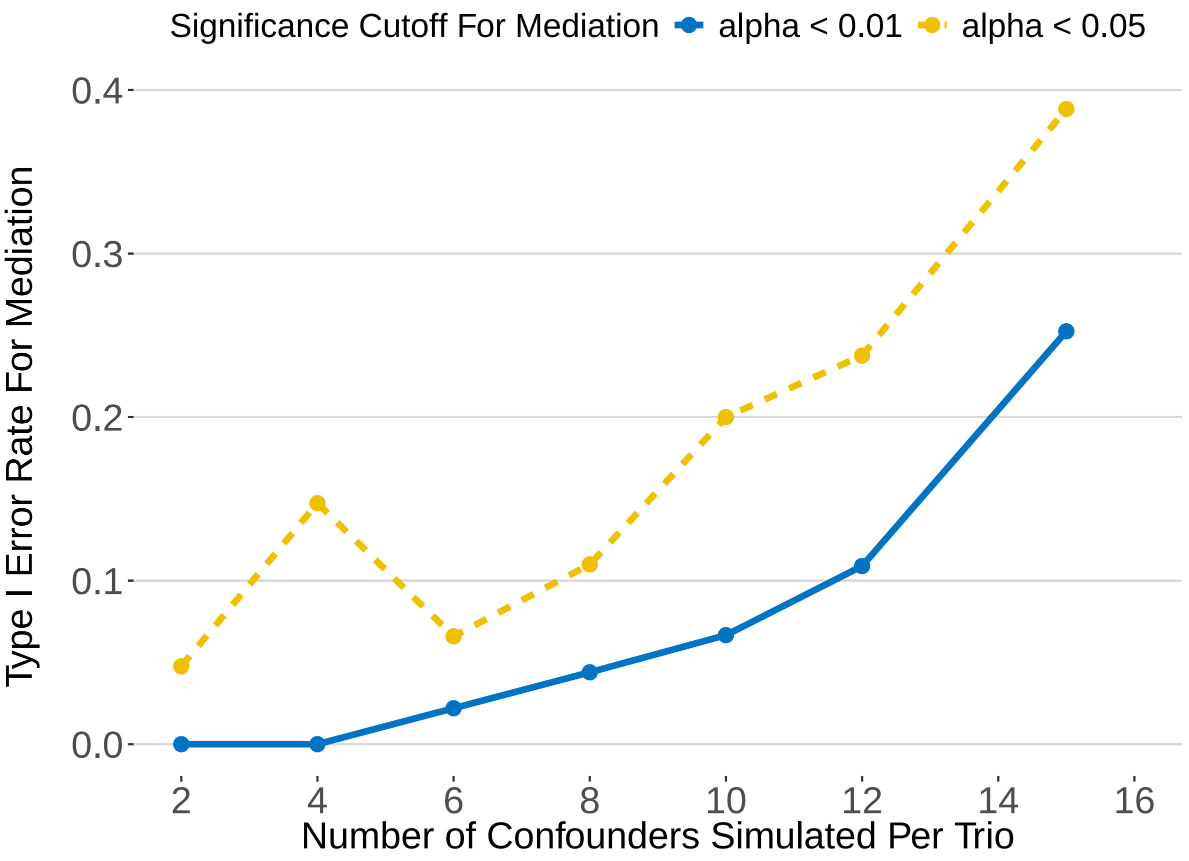

Supplementary Figure 8: Comparison of GMAC and MRGN type I error rates for $T_1 - T_2$ detection under confounding. Results of simulations investigating type I error rate (false mediation) under the null model. Trios were simulated under the null model for each of seven scenarios: 2, 4, 6, 8, 10, 12 or 15 confounders, excluding common child and intermediate variables. The GMAC method was applied to trios in each scenario using CS-q confounder selection.

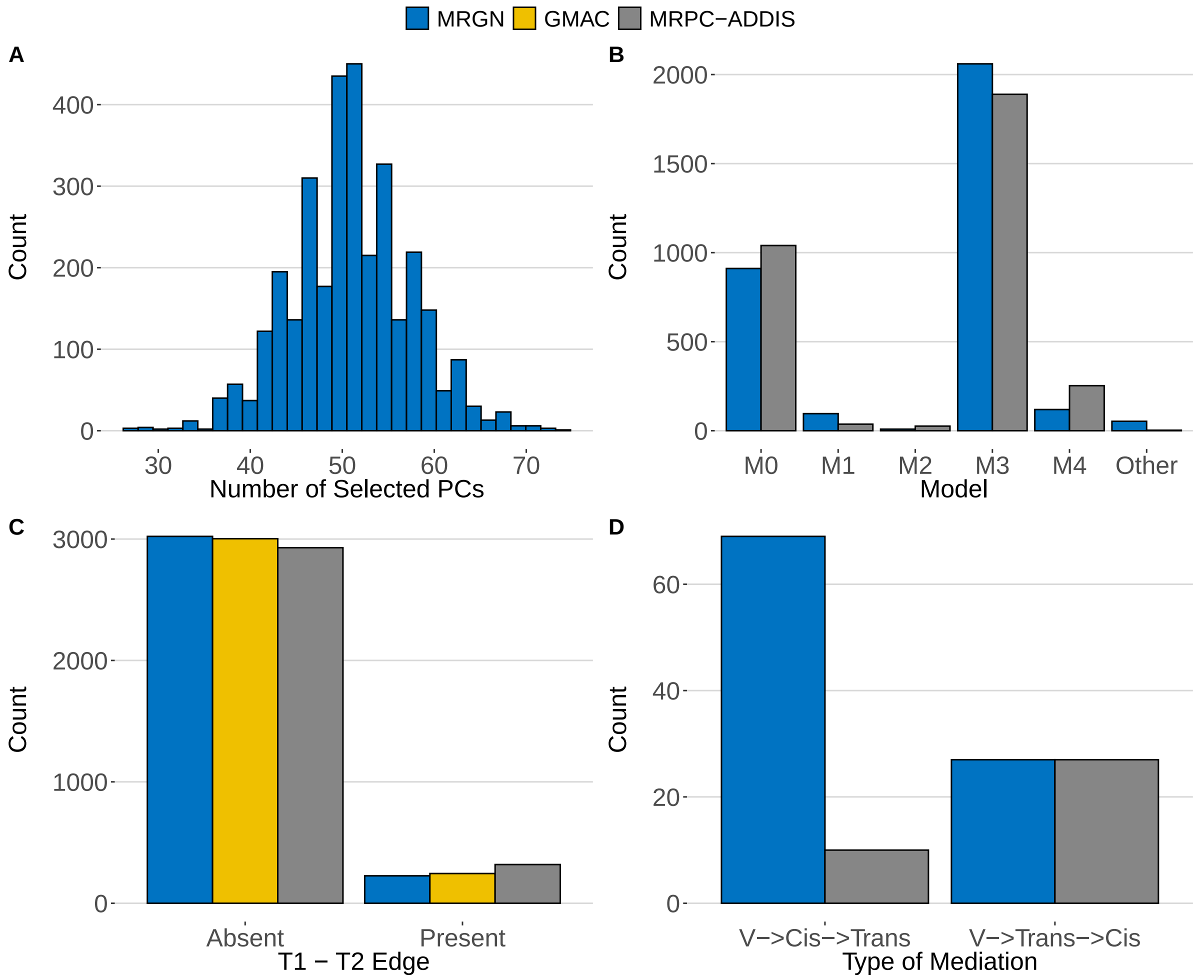

Supplementary Figure 9: Comparative analysis of MRGN with CS-α, MRPC and GMAC on GTEx human whole blood trios.
