# Supplementary material for: Inferring causal networks for genomic trios with high-dimensional confounding variables
Source: Brief Bioinform. 2026 Nov 5;27(6):bbag611. doi: 10.1093/bib/bbag611 (PMC13738103; doi:10.1093/bib/bbag611)
Supplement: Supplementary file 1 [file supplementary_materials_bbag611.zip › Supplementary_Text_bbag611.pdf]

Inferring causal networks for genomic trios with high-dimensional confounding variables

Supplemental Text

Simulation study on model misspecification

In this mini-simulation, we examine the impact of changes in selected confounding variables on the power to detect mediation. Our simulations are based on real whole blood trios from GTEx (version 8) identified as causal mediation using the GMAC method (Yang et al. 2017). Since GMAC is agnostic to the direction of the mediation relationship, these trios may, in reality, be trans-gene mediated ($V \rightarrow T_{trans} \rightarrow T_{cis}$), cis-gene mediated ($V \rightarrow T_{cis} \rightarrow T_{trans}$), or bi-directional: $T_{cis} \leftrightarrow T_{trans}$).

In each simulation scenario, we follow a consistent process to simulate each mediation trio under a specific model of confounding. First, we fit a regression model where the trans gene is predicted by the genetic variant, the cis-gene, and a set of confounding variables. The second step is to simulate new values of the trans-gene under the same model but using the estimated effects from the regression in the previous step. This establishes the confounders in the previous step as the true set of variables confounding the relationship between the cis and trans gene. Moreover, since each trio has been previously identified by the GMAC method to exhibit positive mediation, the estimated effect between the cis and trans gene is significant and establishes causal mediation as the simulation truth.

After simulating the expression of the trans gene under a specific model of confounding using the steps above, we investigate the impact of model misspecification by adding or removing confounders with weak to moderate effects on the edge between the cis and trans gene. This process allows use to determine the robustness of regression based methods like our MRGNtrio method to errors introduced by confounder selection.

1 The Small True Model Simulation

In this scenario we simulate data using a set of confounders and analyze the data using true confounders as well as false positives, such that the inference model is overspecified.

We start by first fitting a regression of the trans gene on the genetic variant (denoted V), cis gene (denoted T_1), and set of confounding variables chosen by the CS-q selection (denoted **U**) method to obtain the estimated effects for each covariate eq.(1).

$$T_2 = \beta_0 + \beta_{21}V + \beta_{22}T_1 + \Gamma U + \epsilon \qquad (1)$$

We then simulate the trans gene using these estimated effects in eq.(2):

$$T_2^* = \hat{\beta}_0 + \hat{\beta}_{21}V + \hat{\beta}_{22}T_1 + \hat{\Gamma}_W \mathbf{W} + \epsilon^*, \qquad (2)$$

where $\mathbf{W}$ is a subset of the most significant confounders from **U**. This subset consists of confounders with p-values below $\alpha = 0.001$ in the Wald tests from regression in eq.(1). The errors ϵ^* are sampled from $N(0, \hat{\sigma}_\epsilon^2)$.

We then attempt to recover the mediation model using eq. (3):

$$T_2^* = \beta_0 + \beta_{21}V + \beta_{22}T_1 + \Gamma_1\mathbf{W} + \Gamma_2\mathbf{M} + \epsilon, \qquad (3)$$

where **M** represents the additional confounders in **U** that are false positives and wrongly included.

2 Large True Model Simulation

This scenario represents a case where the inference model is underspecified and includes all the confounders with strong effects but omits many true ones with weaker effects.

We first fit the regression model given in eq. (4) to obtain coefficient estimates:

$$T_2 = \beta_0 + \beta_{21}V + \beta_{22}T_1 + \Gamma_1\mathbf{U} + \Gamma_2\mathbf{Z} + \epsilon, \qquad (4)$$

where **Z** represents a set of additional covariates. We obtain the set **Z** by uniformly sampling 1 – 20 additional confounders from the pool of potential confounding variables, which generally have weak effects. We simulate the trans gene using estimated effect sizes:

$$T_2^* = \hat{\beta}_0 + \hat{\beta}_{21}V + \hat{\beta}_{22}T_1 + \hat{\Gamma}_1\mathbf{U} + \hat{\Gamma}_2\mathbf{Z} + \epsilon^*. \qquad (5)$$

We then attempt to recover the simulation truth using only **U**:

$$T_2^* = \beta_0 + \beta_{21}V + \beta_{22}T_1 + \Gamma_1\mathbf{U} + \epsilon. \qquad (6)$$

3 Correct Model Simulation

The previous simulations seek to understand the impact of model misspecification on the power of the conditional test between the cis and trans genes conditioned on the genetic variant and confounder covariates. In this scenario, we use the same model for simulation and inference to assess the stability of the inference when the inference model is correct.

4 Small Inference Model Simulation (SIMS)

In this scenario, we again use the model from eq. (1) to generate the trans gene. The inference model, however, includes only the top few confounding variables identified by MRPC in Kvamme et al. (2022). This setup represents extreme model underspecification, allowing for a direct comparison between the power of the MPRC analysis and our current analysis using MRGN.

References

Kvamme, Jarred, Md Bahadur Badsha, Evan A Martin, Jiayu Wu, Xiaoyue Wang, and Audrey Qiuyan Fu. 2025. “Causal network inference of cis-and trans-gene regulation of expression quantitative trait loci across human tissues.” *Genetics* 230 (2): iyaf064.

Yang, Fan, Jiebiao Wang, Brandon L. Pierce, Lin S. Chen, and GTEx Consortium. 2017. “Identifying cis-mediators for trans-eQTLs across many human tissues using genomic mediation analysis.” *Genome Research* 27 (11): 1859–71.
